# Sustained Incompatibility between MAPK Signaling and Pathogen Effectors

**DOI:** 10.3390/ijms21217954

**Published:** 2020-10-26

**Authors:** Julien Lang, Jean Colcombet

**Affiliations:** 1 Institute of Plant Sciences Paris Saclay (IPS2), CNRS, INRAE, UEVE, Université Paris-Saclay, 91405 Orsay, France; 2 Institute of Plant Sciences Paris Saclay (IPS2), CNRS, INRAE, UEVE, Université de Paris, 91405 Orsay, France; jean.colcombet@inrae.fr

**Keywords:** Mitogen-Activated Protein Kinases (MAPK), effectors, plant pathogens, ETI, PTI, phosphocode

## Abstract

In plants, Mitogen-Activated Protein Kinases (MAPKs) are important signaling components involved in developemental processes as well as in responses to biotic and abiotic stresses. In this review, we focus on the roles of MAPKs in Effector-Triggered Immunity (ETI), a specific layer of plant defense responses dependent on the recognition of pathogen effector proteins. Having inspected the literature, we synthesize the current state of knowledge concerning this topic. First, we describe how pathogen effectors can manipulate MAPK signaling to promote virulence, and how in parallel plants have developed mechanisms to protect themselves against these interferences. Then, we discuss the striking finding that the recognition of pathogen effectors can provoke a sustained activation of the MAPKs MPK3/6, extensively analyzing its implications in terms of regulation and functions. In line with this, we also address the question of how a durable activation of MAPKs might affect the scope of their substrates, and thereby mediate the emergence of possibly new ETI-specific responses. By highlighting the sometimes conflicting or missing data, our intention is to spur further research in order to both consolidate and expand our understanding of MAPK signaling in immunity.

## 1. Introduction

The plant immune system and its evolution is best described by the zig-zag model, in which plants and pathogens conduct constant warfare giving rise to different layers of plant susceptibility and resistance [1]. This model actually resumes and generalizes Flor’s gene-for-gene concept, according to which for each gene that conditions resistance (R) in the host, there is a corresponding gene that conditions pathogenicity in the parasite [2]. 

The first branch of the zig-zag system relies on the extracellular recognition, by the plant, of Pathogen-Associated Molecular Patterns (PAMPs) like flagellin or chitin. The transmembrane proteins allowing PAMP sensing are Pattern Recognition Receptors (PRRs) [3], and upon the binding of their cognate ligands, PRRs initiate intracellular signaling and responses, culminating in PAMP-Triggered Immunity (PTI) [4]. Although often not considered as being R genes, PRRs provide partial resistance and can even confer full resistance when transferred to other plant species [5].

To counter the first layer of defense, pathogens secrete effectors, extracellularly or directly within the cytoplasm of the plant hosts, which can negatively interfere with PTI. As a result, plants are not able to defend themselves anymore and fall into the state of Effector-Triggered Susceptibility (ETS). 

In response, the plants evolve mechanisms to revert ETS. Fitting the gene-for-gene concept, the role of an R protein is to recognize the actions of a pathogen effector (qualified for this reason as avirulent or Avr), and thereupon to elicit new defense responses which will consolidate a novel layer of immunity: the Effector-Triggered Immunity (ETI). Over the past few years, progress in the field of plant–pathogen interactions has revealed that the recognition of intracellular effectors is ensured by a typical group of R proteins—the Nucleotide-Binding Leucine-Rich Repeat Receptors (NLRs). Moreover, different mechanisms of pathogen recognition have been identified. Either the recognition is through direct interactions between NLRs and effectors, or the recognition is indirect when the NLRs are activated by modifications in the effector targets. In this latter case, we refer to the guard or the decoy hypotheses, depending on the primary roles in immunity of the modified targets [6,7]. The plant family of NLRs comprises two major classes which differentiate in their N-terminal domains, as follows: the Toll Interleukin-1 Receptor NLR (TNL) class and the Coiled-Coil NLR (CNL) class. Despite significant advances in the past years, the ways NLRs transduce ETI signaling and activate defense responses remain poorly understood [6,7]. A classical view emanating from the initial study of Aarts et al. [8] considers that CNL-mediated ETI and TNL-mediated ETI signalings act in independent pathways, mediated by two distinct master regulators: the integrin-like Nonrace-specific Disease Resistance 1 (NDR1) and the lipase-like Enhanced Disease Susceptibility 1 (EDS1), respectively. However, more recent findings have refined this general assumption. For instance, some CNLs are needed as helpers of some TNLs to establish ETI responses [9]. Conversely, EDS1 was shown to be instrumental not only in TNL-mediated ETI signaling but also in CNL-mediated ETI signaling, notably by buffering the Salicylic Acid (SA) sector of defense [10,11]. Finally, diverse CNL receptors, such as RPP7, RPP8 and ZAR1, turned out to mediate defense responses irrespective of NDR1 [12].

The distinction between PTI and ETI in the zig-zag model also raises challenging questions regarding the dynamic articulation of these two immunity layers, as well as the nature of the defense responses they elicit. The similarities and specificities of PTI and ETI have been extensively addressed in recent reviews [13,14]. The current conception suggests that there is a strong overlap between the downstream responses of PTI and ETI, although these responses seem stronger and more prolonged during ETI compared to PTI. Hypersensitive Responses (HR) associated with cell death and Systemic Acquired Resistance (SAR) appear also as typical features of ETI. In addition, several lines of evidence indicate that PTI and ETI share some common signaling components, even if the degree of activation or the coordination of these components might differ, allowing for instance rather compensatory effects in ETI, and rather synergistic effects in PTI. Due to these differences, ETI is considered as a more robust phenomenon than PTI [15].

Mitogen-Activated Protein Kinase (MAPK) cascades form one of the signaling components mentioned above that are involved both in PTI and ETI. Canonically, an MAPK cascade comprises an MAP3 Kinase (MAP3K), an MAP2 Kinase (MKK) and an MAPK (MPK), which activate each other in a serial manner, so that active MPK can ultimately phosphorylate its substrates and implement appropriate cellular responses. The activation of MPKs by MKKs occurs through the dual phosphorylation of the conserved TXY motif. In plants, MAPK cascades are encoded by large multigenic families, a feature contributing to the complexity of these signaling networks. Homology analysis in *Arabidopsis thaliana* identified 80 MAP3Ks spread in three subfamilies (MEKK, ZIK and Raf), 10 MKKs split in four clades and 20 MPKs also distributed in four clades [16]. Different roles for MAPK cascades have been documented in developmental processes as well as in responses to abiotic and biotic stresses [17]. In the immunity context, two MAPK cascades have been particularly well studied. Both MAPKKK3/5-MKK4/5-MPK3/6 and MEKK1-MKK1/2-MPK4 cascades are thus rapidly activated upon PAMP perception [18,19,20], and these rapid but transient activations are pivotal for the transcriptional reprograming that is characteristic of PTI [21]. Interestingly, in the past decades, activations of MPK3/6 have also been observed during ETI in different plant species, with a delayed and sustained kinetic response, and specific contributions to the ETI responses of some MAPK components have been uncovered.

In this review, we will focus on these latest findings revolving around the roles of MAPK signaling during ETI. We searched the literature and gathered different data concerning (1) the MAPKs as targets of pathogen effectors, (2) the dynamics and the regulation of MAPK activations during ETI, (3) the functions of MAPKs during ETI, and (4) the substrates of MAPKs in ETI responses. Our concerns were to highlight the differences in the modes of action of MAPK signaling between PTI and ETI, and to point out the areas which might deserve further exploration.

## 2. MAPKs as Targets of Virulent and Avirulent Effectors

**Figure 1:**
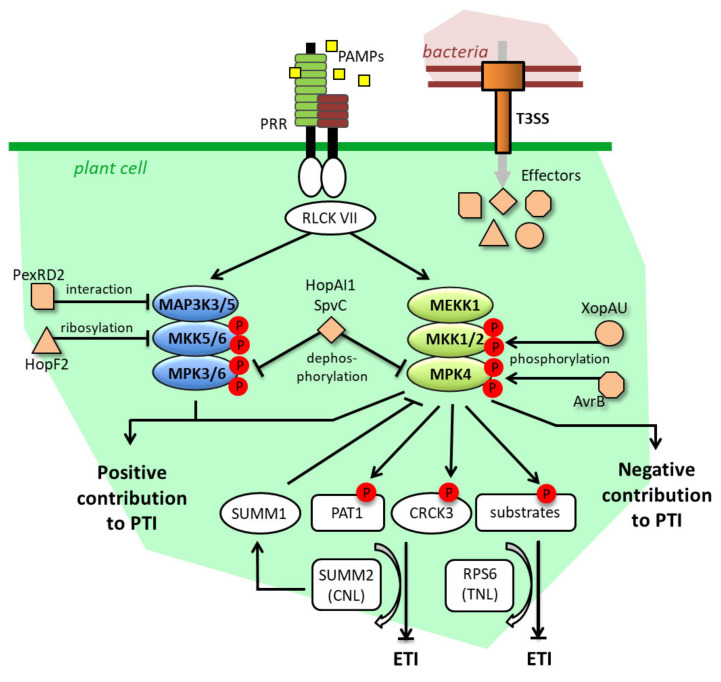
Pathogen effector targeting and guarding mechanisms of MAPK signaling. Effectors target MAPK signaling at different levels and through different molecular modifications. These targetings promote virulence either through the inhibition of the positive contribution to PTI of MAPK, or through the promotion of the negative contribution to PTI of MAPK. In parallel, the MEKK1-MKK1/2-MPK4 cascade is guarded by both the CNL SUMM2 and the TNL RPS6 through the monitoring of various MPK4 substrates, which allow the fine-tuning of ETI responses. An ETI-reinforcing loop, involving the MPK4-interacting SUMM1 protein, also exists between SUMM2 and MPK4. For detail, see main text. T3SS: Type 3 Secretory System, PRR: Pattern Recognition Receptor, RLCK VII: Receptor-Like Cytoplasmic Kinase of group 7.

Plant pathogens comprise bacteria, viruses, oomycetes, fungi and herbivores, and a number of these organisms are known to be able to secrete effectors which facilitate the colonization of their hosts [22]. For instance, different bacterial strains of the model phytopathogens *Pseudomonas syringae* and *Xanthomonas* spp. have a priori arsenals of several dozens of effectors they can inject into the plant cells through a Type-III Secretory System (T3SS) [23,24]. Several strategies deployed by these effectors to promote infection through the inhibition of PTI responses have been described. Since they are central components of the biotic stress responses, the MAPK cascades are targets of choice for these effectors. To our knowledge, six of them have hitherto been characterized as directly manipulating the MAPK cascades in plants, as follows: *P. syringae* AvrB, HopAI1 and HopF2, *Salmonella typhimurium* SpvC, *X. euvesicatoria* XopAU, and *Phytophtora infestans* PexRD2. If, in general, these effectors increase the virulence of the bacteria that express them, we will also see that they may sometimes be under the surveillance of an NLR, and provoke ETI. Figure 1 depicts in a condensed manner these different molecular mechanisms of MAPK targeting by effectors, and of MAPK guarding by NLRs, which will be discussed more in detail in the following.

Historically first, HopAI1 was shown to directly desactivate MPK6 and MPK3 by dephosphorylating them through a specific phosphothreonine lyase activity. Consistently, strains presenting a loss of function for the HopAI1 gene were less virulent in tomatoes than their wild type (WT) counterparts [25]. Likewise, SpvC, a close homologue of HopAI1 found in S. *typhimurium,* can dephosphorylate MPK6, and thereby fosters the virulence of the pathogen [26]. HopF2 is another effector targeting the MAP3K3/5-MKK4/5-MPK3/6 cascade. By adding ADP-ribose moieties to MKK5, HopF2 blocks its kinase domain, prevents MPK3/6 activations and contributes to the virulence of *P. syringae* in tomato and *A. thaliana* [27]. Remarkably, while it is virulent in the latter two plant species, HopF2 is avirulent in tobacco, suggesting that its actions might fit the gene-for-gene concept in some genotypes and be recognized by some NLR, possibly at the level of the MKK4/5-MPK3/6 cascade. HopF2 also targets BAK1, a coreceptor of different PRRs, thereby impairing PTI ab initio [28]. This strategy that causes an indirect inhibition of the MAPK signaling upstream of its individual components is actually shared by many effectors. As examples, we can cite *P. syringae* AvrPto and AvrPtoB, which also block PRRs like FLS2, EFR and CERK1 [29,30], or the *X. campestris* AvrAC, which dampens PTI just downstream of the PAMP receptors by the uridylation of Receptor-Like Cytoplasmic Kinases (RLCKs) [31].

Concerning the MEKK1-MKK1/2-MPK4 cascade, the fact that the three *A. thaliana* mutants *mekk1*, *mkk1mkk2* and *mpk4* display autoimmune phenotypes associated with dwarfism is coherent with the notions that this MAPK cascade is actually both targeted by some effectors and guarded by some NLR, and that the constitutive immune responses observed in the mutants were provoked by the misfiring of the NLR. This hypothesis was confirmed through a suppressor screen, which identified the suppressor of *mkk1mkk2* phenotypes 2 (SUMM2) as being a CNL receptor. In the same study, authors found that the effector HopAI1 could co-immunoprecipitate with MPK4, and that the expression of HopAI1 triggered defense responses which were abolished in the *summ2* background, strongly suggesting that HopAI1 could desactivate MPK4 in the same manner as it desactivates MPK3 and MPK6 [32], and that this effect was guarded by the CNL SUMM2. In addition to SUMM2, two other suppressors of *mkk1mkk2* phenotypes were characterized. The gene *SUMM1* codes for the MAP3K MEKK2, and is a member of a tandemly duplicated gene family with *MEKK1* and *MEKK3*. As the overexpression of MEKK2 (SUMM1) promotes defense responses in a SUMM2-dependent way, it was first believed that the increased expression levels of *MEKK2* (*SUMM1*) measured in the *mekk1*, *mkk1mkk2* and *mpk4* lines were the cause of SUMM2 activation [33,34]. However, a subsequent study showed that this upregulation was dependent on SUMM2, and that MEKK2 (SUMM1) has the ability to directly inhibit MPK4. These results prompted the creation of a positive feedback model in which active SUMM2 provokes the upregulation of *MEKK2* (*SUMM1*) to decrease MPK4 activity, and reinforces defense responses [35]. SUMM3 corresponds to the Calmodulin-binding Receptor-like Cytoplasmic Kinase 3 (CRCK3). It is a substrate of MPK4 and it interacts with SUMM2 in vivo. Therefore, CRCK3 (SUMM3) appears as a guardee of SUMM2 and it is assumed that SUMM2 can monitor MPK4 activity through the phosphorylation levels of CRCK3 [36]. Interestingly SUMM1, SUMM2 and SUMM3 revert the *mpk4* phenotype partially, and the *mekk1* and *mkk1mkk2* phenotypes totally, suggesting that MPK4 could impede the activation of defense responses independently of MEKK1, MKK1/2 and SUMM2, in a manner which still needs to be fully clarified.

Extending our understanding of how the MEKK1-MKK1/2-MPK4 cascade is guarded, two new actors were recently identified. The disruption of the gene *PAT1*, which encodes a member of the mRNA decapping machinery, results in a SUMM2-dependent autoimmune phenotype. As a substrate of MPK4 and an interactor of SUMM2, PAT1 might allow SUMM2 to trigger defense responses through the monitoring of MPK4 activity, similarly to what is known for CRCK3. The non-occurrence of *PAT1* in the *mkk1mkk2* suppressor screen might also be explained by the fact that, while *CRCK3*’s loss of function stops the activation of SUMM2, the loss of function of *PAT1* promotes SUMM2-mediated defense responses [37]. Using an alternative suppressor screen based on the dominant negative effect of the N-terminal part of MEKK1, the TNL RPS6 was revealed as a positive regulator of *mpk4* and *mekk1* phenotypes [38]. The involvement of the TNL RPS6 is in agreement with the previous findings that the *mpk4* and *pat1* phenotypes are partly dependent on EDS1 [37,39]. However, how the CNL SUMM2 and the TNL RPS6 are articulated is not clear. The possibility that SUMM2 could act as a helper of RPS6 was excluded because SUMM2 is not required for the capacity of RPS6 to recognize the effector HopA1 (different from HopAI1), and therefore the hypothesis that RPS6 presumably guards proteins other than CRK3 and PAT1 is preferred for the moment [38]. On the other hand, the role of RPS6 in the recognition of the effector HopAI1 has not been tested yet, and analysis of the double mutant *summ2rps6* might deliver valuable information in the future.

The impacts on the MEKK1-MKK1/2-MPK4 cascade of *P. syringae* AvrB and the *X. euvesicatoria* XopAU effectors are a priori counter-intuitive. Indeed, instead of inhibiting it, both of them stimulate its activation. In association with the chaperone HSP90, AvrB can interact with MPK4 and induce its dual phosphorylation on the TXY motif. Whether this phosphorylation is direct, through a kinase activity of AvrB, or indirect through for instance the increased activation of MKK1 and MKK2, has not been determined [40]. In contrast, XopAU possesses a kinase activity that completes the direct phosphorylation of MKK2 on activation sites, suggesting that XopAU may activate the whole MEKK1-MKK1/2-MPK4 cascade [41]. The advantages that bacteria would gain from expressing effectors that activate MAPK signaling remain obscure, but the apparent ambivalent role played by MPK4 in PTI and basal resistance, contributing both to positive and negative responses [42,43], might in part account for this phenomenon. In the same order of idea, while preparing this manuscript, it was reported that the potato MKK1 is a negative regulator of PTI, which is targeted and stabilized by the effectors PITG20303 and PITG20300 from the Irish potato famine pathogen *P. infestans* in order to promote infection [44].

To conclude this part, we will also mention the *P. infestans* effector PexRD2, which can interact with and inhibit the kinase domain of the MAP3Kε [45]. In tobacco and tomato, MAP3Kε, acting upstream of WIPK and SIPK (homologues of MPK3 and MPK6, respectively), is a positive regulator of defense, notably by promoting cell death in response to various *Pseudomonas* and *Xanthomonas* effectors [46]. Consistently, the impairment of MAP3Kε functions, either by silencing or by PexRD2 expression, results in limited cell death, decreases in MAPK activity and enhanced plant susceptibility [45].

## 3. MAPK Activities during ETI: General or Peculiar?

**Figure 2:**
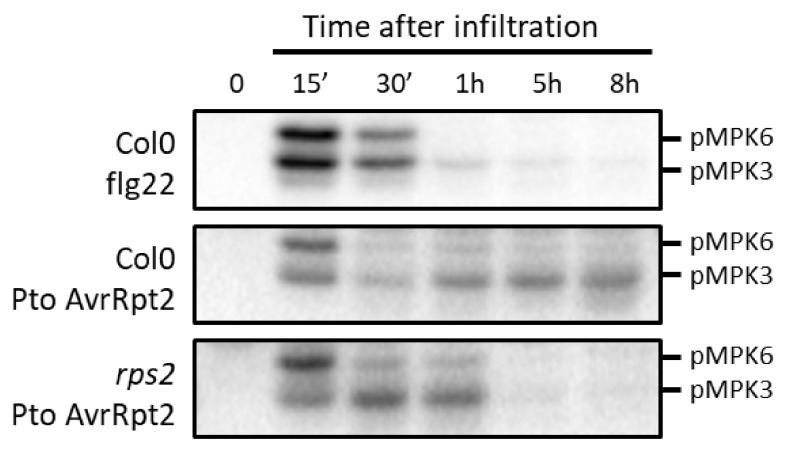
Transient versus sustained activations of MPK3/6 in response to PAMPs and effectors, respectively. Inspired by Tsuda et al. [49]. The leaves of six-week-old *Arabidopsis* Col0 and *rps2* plants (grown in short day conditions in soil) were infiltrated with 1mM of the PAMP flg22 or with Pto expressing AvrRpt2 (OD600 nm = 0.015) for different periods of time, and MPK3/6 activities were measured by immunobloting using an anti-pTpY antibody recognizing the dual phosphorylation of the TXY motif.

**Figure 3:**
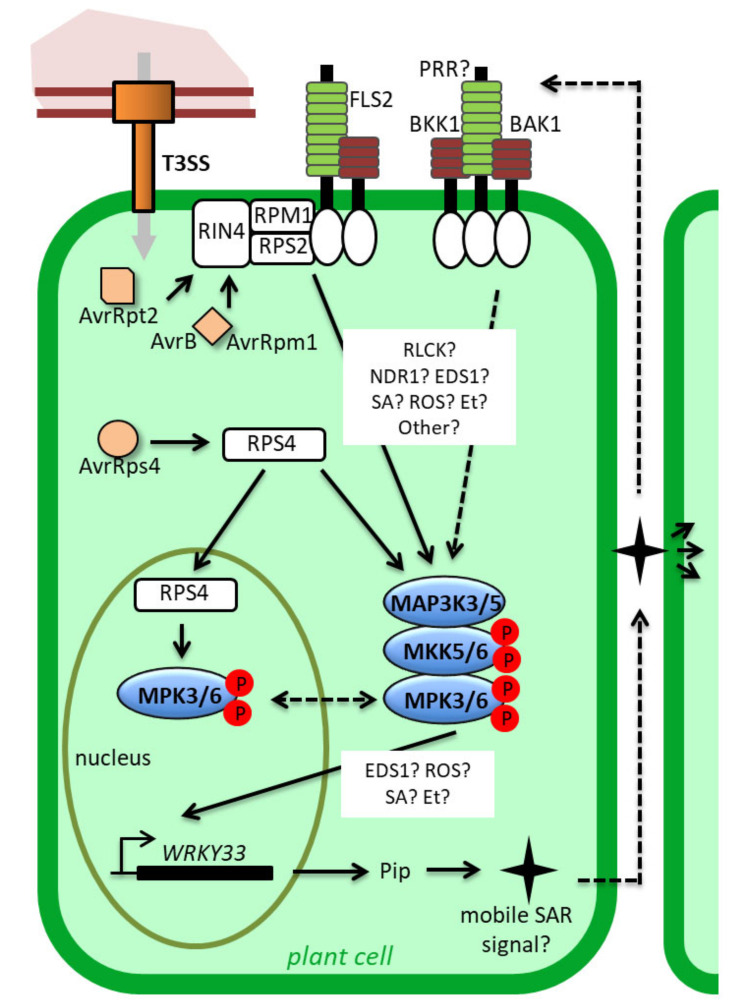
Representation of the demonstrated and putative mechanisms triggering and regulating sustained MPK3/6 activities in ETI. MPK3/6 are activated upon recognition of the effectors AvrRpt2, AvrB and AvrRpm1 by the CNLs RPS2 and RPM1 in the plasma membrane. The roles of RLCKs, NDR1 and EDS1, as well as SA, ROS and Et signaling, in this process remain to be fully elucidated. MPK3/6 might also be activated upon the recognition of AvrRps4 by the TNL RPS4. This activation might require or lead to the nuclear translocation of MPK3/6. Once activated, MPK3/6 induces the expression of WRKY33 in a manner which may be dependent on EDS1, as well as ROS, SA and Et signaling. At last, WRKY33 expression results in the synthesis of a mobile SAR signaling which is supposed to be recognized by an unknown PRR to trigger a new wave of MPK3/6 activation, thereby consolidating a sustained pattern of activation. For detail, see the main text. Broken arrows and question marks indicate hypothetical actions or components. T3SS: Type 3 Secretory System, PRR: Pattern Recognition Receptor, RLCK: Receptor-Like Cytoplasmic Kinase, Pip: pipecolate.

Aside from being targets of effectors, MAPK signalings are also actively involved in ETI responses. In the past few years, several studies focusing on the consequences of effector–NLR recognition have reported MAPK activations, exemplified in Figure 2, which, unlike PTI, were not transient (a few minutes) but delayed and prolonged (several hours). Yet important questions remain to be settled to decide whether these activations constitute a general feature of ETI signaling or are peculiar phenomena dependent on specific effector–NLR pairs. Which MAPK cascades are activated? Which ETI signalings are responsible for these activations? How are the sustained activations maintained? These are the points we will now discuss and which are summarized in Figure 3.

In tobacco, it was shown more than 20 years ago that leaf infection with Tobacco Mosaic Virus (TMV) causes a delayed and long-lasting (several hours) activation of WIPK and SIPK, in a manner which requires the TNL N receptor. Moreover, these activations are correlated with an increase in WIPK protein levels and an SA-dependent and systemic increase in the levels of WIPK transcripts [47]. The activation and expression increase of WIPK (starting about 4 h post treatment and lasting at least 4 h) were also observed in tobacco leaves exposed to the fungal toxin cryptogein from *Phytophotora cryptogea*, but this time no dependency on an NLR gene was demonstrated [48].

Similarly in *Arabidopsis*, sustained activations of MPK3/6 were early observed in response to the fungal pathogen *Botrytis cinerea*, without yet evidence that these activations were linked to an NLR gene [50,51,52]. It was only later, with the use of the model phytopathogen *P. syringae*, that the relation between MAPK activation and ETI responses could be substantiated. Even if sustained activations of MPK3/6 in response to *Pseudomonas* strains expressing avirulent effectors were already described beforehand in *Arabidopsis* [53,54], the cornerstone study on the topic dates from 2013. In this one, Tsuda et al. [49] established two important points: (1) MPK3, MPK6 and MPK4 can be activated in a sustained fashion (for at least 10 h) in response to the effector AvrRpt2, whether this is secreted from the *P. syringae* pv *tomato DC3000* (Pto) strain or directly expressed under the control of a steroid-inducible promoter in transgenic plants. Furthermore, the activation concerns mostly MPK3, although the protein levels of MPK3 remain unchanged. (2) The pattern of MAPK activation is abolished in the *rps2* mutant, which is defective for the CNL receptor of AvrRpt2, thereby demonstrating that this activation really depends on the recognition of the effector. By contrast, strains carrying an empty vector or mutated in the T3SS are both unable to activate the MAPKs, while a strain expressing the effector AvrRps4, which is recognized by the TNL RPS4, can induce a weak activation of MPK3 and MPK6, but unfortunately in this case, MAPK activations were not measured in the *rps4* background.

Since then, a couple of studies have brought supplementary information. For instance, Su et al. [55] analyzed the activations of MAPKs upon inoculation with Pto strains expressing the effectors AvrRpm1, AvrB, AvrRpt2 and AvrRps4 at 1.5 h, 4.5 h and 9 h post infiltration, as compared to a control condition consisting of inoculation with a Pto strain carrying an empty vector. As AvrRpm1 and AvrB are recognized by the CNL RPM1, AvrRpt2 by the CNL RPS2, and AvrRps4 by the TNL RPS4, the objective was to investigate whether MAPK activations were general for the ETI process, or were restricted to some categories of effector–NLR recognition. The authors detected a strong activation of all MPK3, MPK4 and MPK6 1.5 hpi, likely corresponding to PTI responses. At later timepoints they observed the strong activation of MPK3 in all cases but the control, a mild activation of MPK4 in response to AvrRpm1, AvrB and AvrRpt2 at 4.5 hpi, and almost no activation for MPK6, suggesting that sustained activations of MAPKs, and especially MPK3, can be indeed provoked by different effectors and mediated both by CNLs and TNLs. However, it is important to note that in these experiments, the protein levels of the MAPKs were not quantified, nor were the plant mutants tested for the corresponding NLR receptors. Besides, we find in the literature conflicting results regarding the general activation of MAPKs during ETI. As corroborating elements, there is the confirmation that infiltration with a *P. syringae* pv *maculicola* ES4326 (Psm) strain expressing the effector AvrRpm1 does cause a prolonged activation of MPK3 and MPK6 [56]. There is also the observation that the activation, under moderate humidity conditions, of the TNL receptor SSI4, through a gain-of-function mutation, leads to the constitutive activation of MPK3/6 [54]. On the other hand, a recent study using transgenic plants expressing AvrRps4 under the control of a steroid-inducible promoter could not detect any MAPK activation in response to the effector, even at late timepoints [57]. Furthermore, the fact that the overexpression of PAD4, a co-regulator of the EDS1 defense pathway, does not provoke MAPK activation was interpreted as evidence that sustained MPK3/6 activities are not a characteristic of ETI mediated by TNLs [58].

If the contrasting data mentioned above might be partly explained by experimental differences (e.g., different plant species, different infiltration protocols, or different plant ages), they nonetheless demand further clarification. For instance, the activation of MPK3 in ETI mediated by the NLRs N and RPS2 is undoubted, but the question of whether this activation is accompanied by an increase in MPK3 expression is still debated. At the protein level, the answer was yes for tobacco [47] and no for *A. thaliana* [49]. Furthermore, at odds with the last result, transcriptomic and translatomic analyses indicate that both *MPK3* transcription and translation are upregulated in *Arabidopsis* in response to AvrRpt2 [59,60].

The cases of MPK6 and MPK4 are even more problematic. If all the studies claim that the recognition of the effectors results in an activation of both MPK3 and MPK6, the available data display inconsistent patterns of MPK6 activation, which may be qualified as periodic in some cases [49], or even as negligible in other cases [55]. During PTI, concomitant and similar activations of MPK3 and MPK6 within the MAP3K3/5-MKK4/5-MPK3/6 cascade are well characterized [4,17]. It is therefore tempting to extrapolate the same mechanisms for ETI, especially since it has been recently demonstrated that MKK4 and MKK5 largely contribute to sustained MPK3/6 activations in response to *B. cinerea* [61]. Nonetheless, as long as no knock-out mutant for MAP2K or MAP3K is analyzed in a clear ETI context, it is not totally certain that the effector-mediated sustained activities of MPK3 and MPK6 would function through the same module. It is also possible that sustained MPK3 and MPK6 activities are somehow, and to a certain extent, obtained through independent pathways. Interestingly, it was shown that the expression of constitutively active forms of *Arabidopsis* MKK4 or parsley MKK5 results in strong sustained activations of MPK6, but mild sustained activation of MPK3 [49,62], which is the opposite of what is observed with Pto-AvrRpt2 infiltration, raising the possibility that other factors than MKK4/5, or additional factors to MKK4/5, are involved in sustained MPK3/6 activations during ETI. For instance, it is already known that the MAP2Ks MKK7 and MKK9 act upstream of MPK3 and MPK6 in the developmental process and abiotic stress response, respectively [63,64]. Concerning MPK4, some data tend to indicate that the protein could be activated during ETI [49,55]. Yet studies clearly addressing the question are missing. In this regard, it is noteworthy that the expression of AvrRpt2 has the indirect effect of desentisizing MPK4 activation in response to PAMPs, by downregulating or inhibiting MKK1 and MKK2 [65]. This suggests that, in actual fact, the recognition of AvrRpt2 prevents activation of the MKK1/2-MPK4 cascade, but here again there is no evidence that MPK4 activation during ETI would follow this canonical module.

In addition to AvrRpt2 and the fungal effector recognized by the NLR N, sustained MAPK activations were recorded in response to AvrRpm1, AvrB and AvrRps4. Similar activations caused by AvrRpt2, AvrRpm1 and AvrB might make sense because the three effectors target the same host protein, RIN4, that interacts with and is guarded by the two CNLs RPM1 and RPS2 at the plasma membrane [6]. The recognition of the effectors might therefore trigger similar molecular mechanisms, such as rearrangement and/or modifications of plasma membrane protein complexes, leading to the activation of MAPKs in a way reminiscent of what is observed in PTI with PRRs (Figure 3). Consistent with this idea, it was shown that RPM1 and RPS2 could co-immunoprecipitate with the PRR FLS2 [66]. In tobacco, it was also discovered that an RLCK acts at the plasma membrane, at the interface between the active CNL Prf, involved in the recognition of AvrPto effectors, and the MAP3Kα [67], providing thereby a mechanism for MAPK activation comparable to what is known to proceed during PTI responses [68]. Regarding AvrRps4, the question of how MAPKs might be activated is more enigmatic. AvrRps4 is recognized by the TNL RPS4, the nucleocytoplasmic partitioning of which plays important roles for ETI responses. Pre-active forms of RPS4 seem to be mostly nucleocytoplasmic, while active forms accumulate in the nucleus [12,69]. As the mechanisms allowing MAPK activation from nuclear or cytoplasmic inputs are unclear, and as the knowledge about the subcellular localization of MPK3/6 during ETI is limited, it is difficult to envision the way MAPK could be activated by this TNL. Adding to the complexity, it was shown in tobacco that the phosphorylation by SIPK of the co-chaperone SGT1 positively controls the nuclear accumulation of the active TNL N, suggesting that MAPK signaling is not only a consequence of TNL activation, but also contributes to this activation [70]. 

If the molecular processes presiding at the MAPK activations during ETI remain in part elusive, the place of these activations within the whole ETI signaling process is also poorly understood. The proteins NDR1 and EDS1, the phytohormones SA and ethylene (Et), and the Reactive Oxygen Species (ROS) are major regulators of ETI responses [13,15,59], the impacts of which on sustained MPK3/6 activities have been tested using different approaches (Figure 3). Whereas the initial study of Tsuda et al. [49] already demonstrated that the sustained MPK3/6 activations in response to AvrRpt2 are not affected in the *sid2* mutant that is deficient in the synthesis of SA, the role of EDS1 has been inspected only recently. In an attempt to elucidate how SA, EDS1 and MAPK articulate, Cui et al. [58] quantified MPK3/6 activations in response to AvrRpt2 infiltration in the Col0, *eds1* and *sid2* backgrounds. From their results, which showed no differences between the three genotypes, they deduced that sustained MPK3/6 activations act irrespectively of the SA and EDS1 pathways. This is rather surprising, because previous studies indicated that, in RPS2-mediated ETI, both the EDS1 and the MPK3/6 pathways buffer the SA responses [10,11,49], strongly suggesting that the EDS1 and sustained MPK3/6 activities should be in the same pathway. Additionally, the activations of MPK3/6 in the gain-of-function mutant *ssi4* are partly reverted by the *eds1* mutation [54], hinting that actually EDS1 could contribute to MAPK activation in the same ETI signaling branch. Also Cui et al. [58] did not consider the possibility that the MPK3/6 activities could act upstream of EDS1, even if their results are consistent with this possibility. For the future, combined analyses of the *eds1* mutant with MPK3/6 gain-of-function mutants should help in removing these ambiguities. In parallel, based on the expression of the constitutively active form of parsley MKK5, it seems that sustained activations of MPK3/6 depend on Et signaling as well as RBOHD-dependent ROS signaling [62]. Finally, the impact of NDR1 on sustained MPK3/6 activities has not been investigated till now, although this protein, localized at the plasma membrane, is known to be essential for plant resistance against AvrRpt2 and AvrRpm1 effects [8,71,72]. 

To explain how the activities of MPK3/6 are maintained during ETI, two non-exclusive explanations may be advanced. The first hypothesis is that the MAPKs are durably activated by continuous input signals that may originate directly from the pathogen or indirectly from a positive feedback regulation downstream of the MAPK cascade. The second hypothesis is that the mechanisms which usually dampen the MAPK signaling, like desactivation by phosphatases, are blocked during ETI.

Concerning the first hypothesis, the most valid view is that the expression of the avirulent effectors promotes a durable activation of the NLRs, which in turn would feed the MAPK activation in a prolonged manner. However, to fully substantiate this assumption, a better understanding of the NLR turnovers, which is still limited for the moment, would be needed. For instance, during PTI, the inputs generated by PAMPs are rapidly quenched through the internalization and proteasome-addressing of the PRRs [73,74]. Scaffold proteins might also be implicated in this process. In mammals and yeast, these proteins can confer both spatial and temporal regulation of the MAPK modules [75]. By facilitating and/or maintaining the interaction between active MAP2K and MPK3/6, plant ETI-specific scaffold proteins may be therefore responsible for the sustained activations of MPK3/6. Yet little is known about scaffolds in plants, and if RACK1 has been characterized as a scaffold protein for the MEKK1-MKK4/5-MPK3/6 module, its role seems to be restrained to rapid and transient activation in response to some bacterial proteases [76]. In the future, the identification of MAPK scaffolds acting in response to avirulent effectors would represent a big step forward for the understanding of ETI responses and MAPK functioning. On the other hand, it was nicely demonstrated in a recent paper that Pipecolate (Pip) could activate MPK3/6 in a way that depends on the PRR co-receptors BAK1 and BKK1, and that conversely sustained MPK3/6 activities lead to an increase in Pip contents [56]. As Pip is a Lys catabolite which strongly accumulates in infected leaves, as well as in distal uninfected leaves at the onset of SAR, the authors built a model in which sustained MPK3/6 activities are obtained through a positive regulatory loop driven by a mobile SAR signaling molecule emanating from Pip, and recognized by receptor complexes including BAK1 and BKK1 (Figure 3). In support of this model, they showed that upon AvrRpt2 infection, the sustained MAPK activations were lost in knock-out lines for the genes *ALD1* and *FMO1* that encode critical steps in the Pip-mediated SAR pathway, as well as in a knock-out line for the gene *WRKY33* that codes for a transcription factor positively controlling the expression of ALD1. Remarkably, they demonstrated that the sustained MPK3/6 activities induced by the expression of a constitutively active form of MKK4 were also compromised in the *ald1*, *fmo1* and *wrky33* backgrounds. This result may indicate that ALD1, FMO1 and WRKY33 are needed for the proper activation of MPK3/6 by MKK4/5, but more likely they suggest that the activation of MAPKs within the MKK4/5-MPK3/6 cascade is transient per se, and that sustained MAPK activation requires in all cases a positive feedback regulation involving SAR components. In line with this is the evidence that EDS1, Et and ROS produced by RBOHD, which may negatively affect the sustained activation of MPK3/6 [54,62], are also involved in SAR [77,78,79], even if their links with the Pip-driven pathway remain to be fully clarified. Taken together, these findings have another important implication, which is that the prolonged MAPK activations we observe in bulk leaf samples during ETI might actually reflect different cell populations characterized over time by distinct patterns of transient MAPK activation/desactivation. Analysis at the cell level of the ETI responses and of the MAPK activities is now conceivable [80,81,82] and should, in the short term, yield interesting new data, notably in terms of confirming the role of the WRKY33-ALD1-FMO1 regulatory loop for the sustained activation of MPK3/6. With this in mind, we must evoke the fact that a previous study using transgenic *Arabidopsis* plants expressing a constitutively active form of the heterologous NtMEK2, which also provokes sustained MPK3/6 activities, could not find any difference in the pattern of the MAPK activations between WT and *wrky33* backgrounds [83], a result that contradicts the report of Wang et al. [56], and somehow mitigates the SAR explanation. 

Concerning the second hypothesis, it was shown that HAI1, a protein phosphatase type 2C, could desactivate MPK3/6, and that the transcriptional upregulation of *HAI1* caused by coronatine (a jasmonate analog secreted by Pto strains) was attenuated after the recognition of AvrRpt2 by RPS2 [84]. Moreover, an analysis of the translatome of *Arabidopsis* leaves revealed that the translation efficiency of *HAI1* was reduced in leaves infiltrated with a Psm strain expressing AvrRpt2 comparatively to mock-infiltrated leaves [60]. As such these two studies illustrate processes that may account for the durable pattern of MPK3/6 activation during ETI, through the stalling of their desactivation. In the same order of idea, it is possible that other negative regulators of MPK3/6 activities, like the phosphatases AP2C1 [85], PP2C5 [86], MKP1 [87] and MKP2 [88], or the E3 ligase PUB22 [89], are also inhibited during ETI, either through decreases in expression levels/rates or through post-translational modifications affecting their enzymatic functions or stability. However, when we sifted through the literature focusing on the transcriptomes and proteomes of *Arabidopsis* during ETI [59,60,90,91], we could not find any particularly promising candidate among those.

## 4. Functions of MAPKs during ETI: Robustness and Cell Death

The functions of sustained MPK3/6 activities during ETI have been mostly investigated through gain-of-function approaches. Inspired by works in mammals, this strategy was originally developed in plants with a constitutively active form of the tobacco MAP2K NtMEK2 (NtMEK2^DD^) that harbors the two phosphomimicking mutations Thr227Asp and Ser233Asp within the activation motif [92]. As the expression of NtMEK2^DD^ under the control of a steroid-inducible promoter results in the sustained activations of the MAPKs SIPK, WIPK and Ntf4, the system could be conveniently used to explore the roles of these during ETI, irrespectively of pathogen infection. It was thus revealed that the sustained activities of SIPK, WIPK and Ntf4 reinforce plant defense responses by positively controlling an HR-like cell death, whose execution is characterized by a disruption of the choloroplast metabolism followed by a light-dependent generation of ROS [92,93,94,95,96]. Consistently with these roles in disease resistance, the silencing of *SIPK*, *WIPK*, *Ntf4*, *NtMEK2* or the upstream *MAP3Kα* and *MAP3Kε* renders the plants more susceptible to TMV, *P. infestans* and different avirulent strains of *P. syringae* [45,94,95,97]. Moreover, the expression of NtMEK2^DD^ leads to the transcriptional upregulation of two genes coding for a 3-hydroxy-3-methylglutaryl CoA reductase and for an L-phenylalanine ammonia lyase, two enzymes known to be involved in the biosynthesis of phytoalexins and SA, respectively [92]. The transcript levels of three *ACS* genes were also found to be increased upon NtMEK2^DD^ expression, while this increase correlated with the strong accumulation of Et, suggesting a role for the sustained activities of WIPK, SIPK and Ntf4 in the implementation of the Et pathway [98].

In *Arabidopsis*, two distinct gain-of-function strategies were designed in order to widen our knowledge about the functions of sustained MPK3/6 activities. The first one is a direct adaptation of the tobacco strategy and consists in the expression, under the control of a steroid-inducible promoter, of the constitutively active forms of the endogenous MKK4 (MKK4^DD^) or heterologous NtMEK2^DD^ and parsley MKK5 (PcMKK5^DD^), leading to rapid and durable activations of MPK3/6 [49,62,99]. In the second case, an original variant of MPK3 (K3CA), harboring two mutations in the acidic residues of the phosphorylation lip (Asp193Gly/Glu197Ala) and presenting a constitutive activity, is stably expressed under the control of its native promoter in the *mpk3* background [100].

Using the NtMEK2^DD^ system combined with loss-of-function analysis, it was possible to show that the sustained activation of MPK3/6 contributes to the accumulation of antimicrobial compounds from the tryptophan-derived indole glucosinolate (IGS) pathway, as well as the defense phytohormone Et, which are both key for resistance against *B. cinerea* [101,102]. The accumulation of IGSs is notably mediated by two transcription factors, ERF6 and WRKY33, which are substrates of MPK3/6, and whose phosphorylations lead to increased stability and transactivation activity, respectively [83,101]. The Et accumulation is achieved through the WRKY33-mediated upregulation of the *ACS2/6* genes and the stabilization of their products by MPK3/6-dependent phosphorylation [51,103,104]. Interestingly, the levels of Et produced in response to *B. cinerea* are drastically higher than the ones measured during PTI. In the context of the plant immune response, this observation argues in favor of a strengthening function for sustained MPK3/6 activities as compared to transient activation. Furthermore, K3CA plants also accumulate the IGS camalexin and the phenylpropanoid scopoletin through the upregulation of genes involved in their synthesis [100], while the conditional expression of PcMKK5^DD^ results in increased levels of several IGS defense metabolites [62].

**Figure 4:**
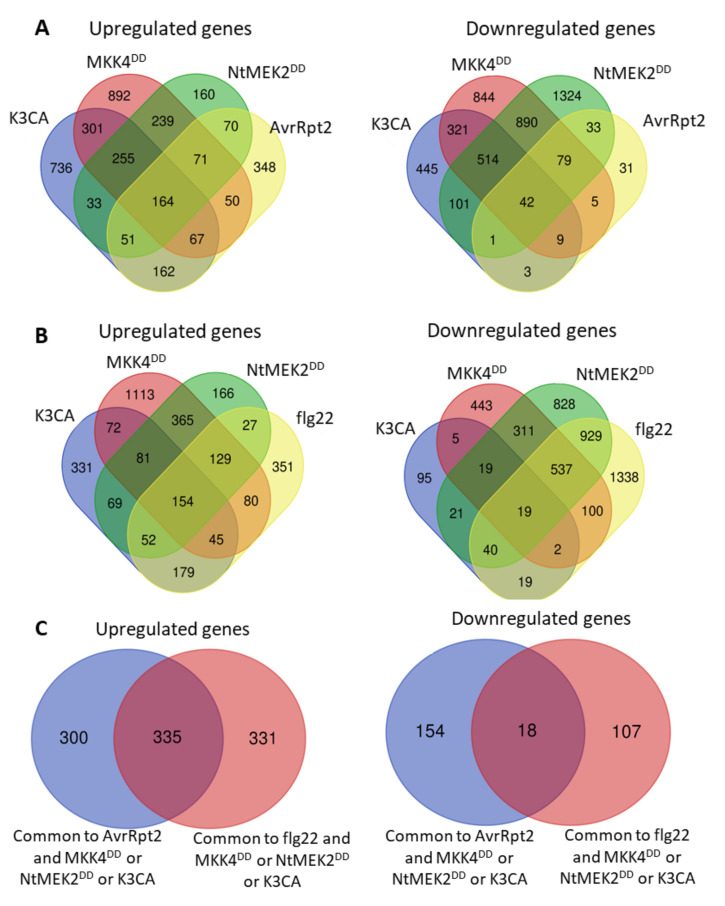
Overlap of the transcriptional reprogramming caused by sustained MPK3/6 activities, ETI and PTI. (**A**) Venn diagrams showing the overlap between genes regulated by sustained MPK3/6 activities (K3CA, MKK4^DD^, NtMEK2^DD^) and expression of the effector AvrRpt2. (**B**) Venn diagrams showing the overlap between genes regulated by sustained MPK3/6 activities (K3CA, MKK4^DD^, NtMEK2^DD^) and the PAMP flg22. The list of flg22-responsive genes was extracted from Frei dit Frey et al. [21]. (**C**) Venn diagrams showing the overlap between genes regulated by sustained MPK3/6 activities (K3CA, MKK4^DD^, NtMEK2^DD^), the expression of the effector AvrRpt2 and the PAMP flg22. Venn diagrams were drawn with the webtool found at http://bioinformatics.psb.ugent.be/webtools/Venn/.

In parallel, the gain-of-function strategies allowed for investigating, at the whole-genome level, transcriptional reprogramming generated by the sustained MPK3/6 activities in *A. thaliana*. The transcriptome analysis with microarray technology of one-month-old leaves, 24 h after the induction of MKK4^DD^ expression, comparatively to mock-treated plants, led to the identification of 2039 upregulated genes and 2704 downregulated genes [49]. Twelve-day-old seedlings of transgenic *A. thaliana*, expressing NtMEK2^DD^ for 6 h, showed, by RNAseq analysis, 1042 upregulated genes and 2984 downregulated genes as compared to seedlings not expressing NtMEK2^DD^ [55]. At last, the aerial parts of one-month-old K3CA plants were subjected to CATMA microarray analysis comparatively to plants expressing a WT form of MPK3, and this permitted the detection of 1769 upregulated genes and 1436 downregulated genes [100]. Noticeably, the comparison of these three transcriptomes to each other, and also with the ETI RNAseq results obtained from one-month-old *A. thaliana* leaves infiltrated for 6 h with a Pma strain expressing AvrRpt2 [60], unveiled a relatively strong overlap (Figure 4A). For instance, about 65% of the upregulated genes (635 out of 983) and 85% of the downregulated genes (172 out of 203) in the ETI context can be retrieved in at least one of the NtMEK2^DD^, MKK4^DD^ or K3CA transcriptomes. Moreover, there are respectively 164 and 42 genes common to the four experiments among the 983 upregulated and 203 downregulated genes found in the ETI RNAseq. Unsurprisingly, a gene ontology analysis revealed in the 65% commonly upregulated genes an enrichment for genes involved in response to bacteria, chitin, hypoxia, incompatible interaction and hormones (SA, jasmonate, Et and abscissic acid), while the 85% commonly downregulated genes were enriched in genes related to response to light (Supplementary Table S1). Taken together, these data support the notion that sustained MPK3/6 activities can significantly direct the expression reprogramming of AvrRpt2-mediated ETI. Moreover, the upregulated genes in the NtMEK2^DD^, MKK4^DD^ and K3CA transcriptomes substantially overlap with the genes upregulated during PTI, which is not the case for downregulated genes (Figure 4B,C), suggesting that transient and sustained MPK3/6 activities might have similar effects on positive transcriptional reprogramming, but discriminate in their abilities to shut down gene expression.

Based on the transcriptomic data, several functions of sustained MPK3/6 activity were unveiled. For instance, a striking feature of the expression profiles from the MKK4^DD^ experiment is their large coincidence with those of SA-responsive genes during AvrRpt2-mediated ETI [49]. Further, this coincidence remains almost the same when MKK4^DD^ is expressed in the *sid2* background, indicating that sustained MPK3/6 activities could buffer the SA sector of defense, i.e., compensate for the usual SA-dependent ETI responses, even in the absence of SA. In support of this, it was shown that the downregulation of the SA-responsive *PR1* gene in the *sid2* background after AvrRpt2 recognition is significantly accentuated in the *sid2mpk3* double mutant, and that, in a correlated way, the double mutant is more susceptible to Pto-AvrRpt2 infiltration than the single mutants [49]. Likewise, the upregulation of *PR1* measured in the K3CA line is reduced in the *sid2* background, without yet retrieving a WT level [100], which provides more evidence that the enhanced and prolonged activity of MPK3 can somehow compensate for SA-dependent gene expression during ETI. Following the same thread of ideas, Wang et al. [56] demonstrated that sustained MPK3/6 activity could induce SAR responses through the transcriptional regulation of the *ALD1* gene in a manner largely independent of SA. In view of all these results, it appears that a key function of the sustained MPK3/6 activities during AvrRpt2-induced ETI is to increase the robustness of the defense responses by securing the redundant regulation of SA signaling. Interestingly, whereas no variation in the SA contents could be detected in plants expressing MKK4^DD^ over 24 h [49], the plants exhibiting a constitutive activity of MPK3/6, like the K3CA lines [100] or the *mkp1* [87] and *ssi4* [54] mutants, do accumulate SA, suggesting that the functional redundancy of sustained MPK3/6 activity might not only act in a pathway parallel to and independent of SA, but might also occur upstream of SA in a somehow energizing fashion. Even if, for correct interpretation, pleiotropic effects should be taken into account in the mutants mentioned above, this possibility is in agreement with the previous observations that NtMEK2^DD^ in tobacco induces the expression of a gene involved in SA production [92], and that PcMKK5^DD^ in *Arabidopsis* promotes the accumulation of several SA precursors [62].

Importantly, it was also noticed that the genes downregulated by sustained MPK3/6 activities are enriched in the genes related to photosynthesis, suggesting that this process might actually be targeted by the MAPKs [55]. In the same study, the analysis with both NtMEK2^DD^ and conditional *mpk3mpk6* double mutant revealed that indeed photosystem II (PSII) is inhibited by sustained, but not transient, MPK3/6 activities, and that, concomitantly to this inhibition, there is a light-dependent ROS accumulation in the chloroplast that precedes cell death. This corroborates previous results indicating that the inducible expression of *NtMEK2^DD^*, *MKK4^DD^* or *PcMKK5^DD^*, as well as the endogenous expression of *K3CA*, provoke HR-like cell death associated with ROS accumulation [62,99,100]. Besides, it was shown that PSII inhibition, light-dependent ROS accumulation and cell death are instrumental in the ETI-mediated resistance of the plants, since the suppression of these phenotypes through the overexpression of a plastid-targeted cyanobacterial flavodoxin leads to a higher susceptibility towards Pto-AvrRpt2 infiltration [55]. Overall, these findings illustrate another way that sustained MPK3/6 activities contribute to the robustness of ETI responses by blocking the perturbations that may come from the carbon fixation metabolism, and by orchestrating a tradeoff between live-and-grow or die-and-resist decisions.

A last aspect highlighted in the NtMEK2^DD^, MKK4^DD^, K3CA and AvrRpt2-ETI transcriptomes is the upregulation of a relatively high number of genes annotated as coding for CNLs and TNLs [49,55,60,100,105]. This observation raises the interesting hypothesis that sustained MPK3/6 activities, downstream of a first effector–NLR recognition, could stimulate the activation of a cohort of subsequent NLRs, offering thereby a new mechanism to reinforce the robustness of ETI responses. Conferring credence to this hypothesis, the disruption of the gene *SNC1* encoding a TNL and of the gene *SUMM2* encoding a CNL can partially revert the constitutive defense responses caused by the enhanced MPK3/6 activities in the *mkp1* [87] and K3CA plants, respectively [100,105]. Since SUMM2 is known to guard the MEKK1-MKK1/2-MPK4 cascade, the latter finding also hints at a possible crosstalk between MAPK modules during ETI. For the future, determining how NLR levels are regulated by sustained MPK3/6 activities, and how they can conjointly impact the strength of the defense responses, should help in gaining a better insight into the ETI processes. 

**Figure 5:**
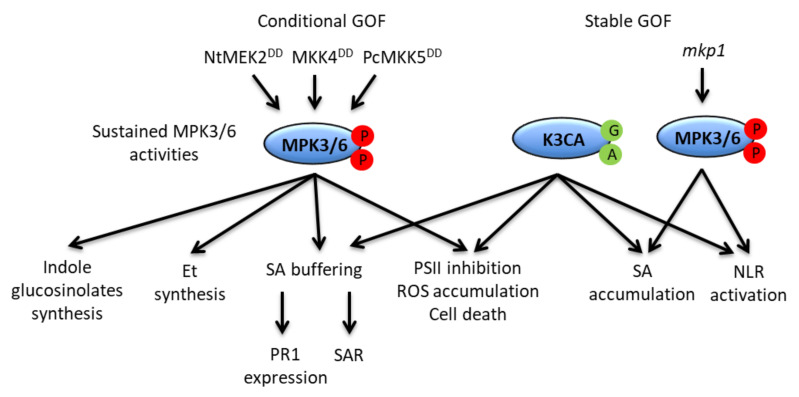
Representation of the functions of sustained MPK3/6 activities. The functions are indicated in connection with the experimental approaches which allowed their elucidation: either conditional gain-of-function (GOF) approaches through the steroid-inducible expression of different constitutively active MAP2Ks, or stable GOF approaches through the endogenous expression of a constitutively active MPK3 (K3CA), and as a result of the *mkp1* mutation. For detail, see the main text.

In conclusion, important and singular functions of sustained MPK3/6 activity have been unveiled thanks to the NtMEK2^DD^, MKK4^DD^, PcMKK5^DD^ and K3CA gain-of-function approaches (Figure 5). However, despite their advantages, these strategies suffer from a certain number of drawbacks that it might be important to recall. For instance, the systems remain artificial, and the pattern of MPK3/6 activities they display does not totally overlap with that caused by AvrRpt2 recognition [49,55,62]. The sustained activation concerns mostly MPK3 in the latter case, and mostly MPK6 in the former one. The sustained activation of MPK4 is also overlooked by these gain-of-function approaches. Furthermore, given that NtMEK2^DD^, MKK4^DD^ and PcMKK5^DD^ act upstream of the MAPKs, they might provoke responses that are independent of sustained MPK3/6 activities. Similarly, the expression of K3CA under its native promoter likely favors pleiotropic effects that could be only distantly related to MPK3 or defense responses. Therefore, the confirmation by a loss-of-function approach of the obtained results is critical, and the development or generalization of new loss-of-function tools, like the nicely-devised chemical–genetically rescued *mpk3mpk6* double mutant system [106] or the new *mkk4mkk5* alleles [107,108], is recommended for the future characterization of the functions fulfilled by sustained MPK3/6 activities during ETI.

## 5. MAPK Substrates during ETI: Is the Temporal Phosphocode Relevant?

To thoroughly elucidate the functions of a MAPK, characterizing its substrates is essential. For this purpose, the usual way to proceed is first to select candidates, then to check, through different in vitro and in vivo approaches, the capacity of the MAPKs to really interact with and phosphorylate these candidates, and finally to perform phenotype complementations with different versions of the substrates, presenting phosphomimicking or phosphodeficient modifications. Several methods were used in the past for the selection of the candidates, such as the yeast two-hybrid screen, peptide/protein arrays, bioinformatic analysis or phosphoproteomics, generating several hundreds of putative MAPK substrates [109]. Harnessing these data grounded a better understanding of the mode of action of the MAPKs, and notably revealed that they are proline-guided serine/threonine kinases, meaning that they preferentially phosphorylate serine or threonine followed by a proline (S/TP sites). Different docking domains mediating the interactions between the MAPKs and their substrates were also proposed [109,110]. Nonetheless, to date, only a few dozen MAPK substrates have been functionally characterized. In the literature, we could find 17 substrates of MPK3/6 as being involved in the plant immunity [70,109,111], and among them just 5 (NtSGT1, AtWRKY33, AtERF6, AtACS6 and AtLIP5) could be associated with sustained activations or ETI responses.

In tobacco, the co-chaperone SGT1 is phosphorylated in vitro by SIPK on the Ser358 of its C-terminal part. Modifications of this phosphorylation site alter the nucleocytoplasmic repartition and activation of the SGT1-interacting TNL N receptor in response to TMV [70]. Curiously, this result suggests that the activation of SIPK is both the cause and the consequence of N-mediated responses. To reconcile the discrepancy, one might reason that the transient activation of SIPK is required for the SGT1-mediated activation of N, which in turn would promote the sustained activation of SIPK to implement fully efficient resistance. In *Arabidopsis*, the transcription factors WRKY33 and ERF6, as well as the Et-biosynthesis ACS6 enzyme, play important roles in resistance against *B. cinerea*, and all three were shown to be substrates of MPK3/6. ACS6 is phosphorylated by MPK6 in vitro. It is also phosphorylated in a delayed manner in vivo, in a MPK6-dependent fashion, upon the conditional expression of *NtMEK2^DD^*, and is rapidly phosphorylated in response to wounding or the PAMP flg22. The targeted mutagenesis of the different S/TP sites in ACS6 revealed that phosphorylations by MPK6 in vitro and in vivo, upon *NtMEK2^DD^* expression or flg22 treatment, occurs on three residues clustered in the C-terminal part of the protein: Ser480, Ser483 and Ser488. Moreover, these three residues are critical for ACS6 stability, because their replacement by non-phosphorylatable alanine residues leads to the increased degradation of the protein when MPK3/6 are active, whereas their replacement by phosphomimicking aspartate residues coincides with higher levels of the protein, even in non-stress conditions [104,112]. Homologous to the C-terminal part of ACS6, the C-terminal part of ERF6 is phosphorylated on Ser266 and Ser269 in vitro by MPK6 and MPK3, and in vivo in a durable manner upon conditional expression of *NtMEK2^DD^*, as well as after infection with *B. cinerea*. Similarly to what was observed for ACS6, the phosphorylation of ERF6 stabilizes the protein, and this stabilization is necessary for the efficient defense response against *B. cinerea* [101,113]. Like ACS6 and ERF6, WRKY33 is sustainably phosphorylated in vivo, likely by MPK3/6, in response to NtMEK2^DD^ or *B. cinerea* infection. WRKY33 harbors five S/TP sites in its N-terminal part (Ser54, Ser59, Ser65, Ser72 and Ser85), and mutations of these five residues into alanine residues impair the phosphorylation of the protein by MPK3/6 in vitro, as well as the induction of the *PAD3* gene and the production of camalexin upon challenge by *B. cinerea*. As the phospho-null mutations affect neither the stability of the protein nor its ability to bind the consensus DNA sequence, it is assumed that the phosphorylation of the five S/TP sites is important for the transactivation capacity of WRKY33 [83]. Lastly, the protein LIP5 is involved in the multivesicular body pathway, which contributes to protein trafficking in many cellular processes. Through different approaches, it was shown that, in response to *NtMEK2^DD^* expression or after infection with the virulent Pto strain, LIP5 is stabilized upon long-lasting phosphorylation, which is surely completed by MPK3/6. Since knock-out *lip5* mutants exhibit a higher susceptibility than WT to avirulent Pto strains expressing AvrB, AvrRpm1 or AvrRpt2, and since, at the same time, these mutant lines do not seem to be affected in their responses to the PAMP flg22 [114], it is possible that the phosphorylation of LIP5 by MPK3/6 has specific functions in response to effector recognition. If true, such a hypothesis would disclose an original link between MAPK, vesicle trafficking and ETI. Therefore, it would be interesting in the future to carry out ETI phenotype complementations with LIP5 versions modified in the six S/TP sites that can be found in the sequence of the WT protein.

Overall, the above substrates illustrate the different mechanisms by which sustained MPK3/6 activities may mediate peculiar functions in an ETI context. The example of LIP5 is of particular interest because it suggests that phosphorylation by MPK3/6 can perform functions which are characteristic of ETI responses. However, these results still need to be confirmed. Besides, the ways the substrates have been characterized do not definitively confirm whether they are specific to sustained or transient MPK3/6 activities. For instance, ACS6 can also be phosphorylated in response to flg22, and the phosphorylation of SGT1 by SIPK seems to occur before the sustained activations of the MAPKs. In the literature, the general conception is that sustained MPK3/6 activities reinforce the effects of transient activities by prolonging them over time [15]. According to this, the scope of substrates targeted by transiently or sustainably activated MPK3/6 would remain unchanged, and it is only the temporal pattern of their phosphorylated forms, and consequently of their effects, that would be extended. 

At this point, we would like to discuss a few data supporting the idea that sustained activations of MPK3/6 can actually impact the scope, not only of their substrates, but also of the phosphorylation sites within these substrates. A key concept here will be that of phosphocode, which stipulates that the function of a protein is modulated by its pattern of phosphorylation [115].

**Figure 6:**
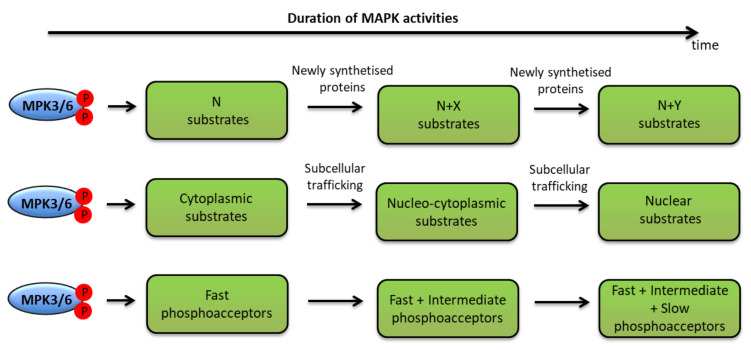
The temporal phosphocode of MPK3/6. Different processes, explaining how durable activities of MPK3/6 might impact the scope of their phosphorylation sites, substrates and functions, are represented: either the scope is modified by newly synthesized substrates (upper line), or by subcellular trafficking allowing new meetings between the kinases and their substrates (middle line), or by the existence of phosphosites which are phosphorylated with different kinetics (lower line). For detail, see the main text.

We previously evoked two scenarios by which sustained MPK3/6 activities could be achieved. In the first one, durable stimulation causes durable activation within the same cell populations. In the second one, a feedback regulation performs successive transient activations in different cell populations. Either way, one may conceive of processes that are able to affect over time the scope of the phosphorylations completed by MPK3/6 (Figure 6).

For instance, along the ETI responses, new substrates might be synthesized or made available through subcellular trafficking. The nucleocytoplasmic shuttling of MAPKs, and especially of MPK3, seems crucial for their function [52,116,117,118]. Therefore, different pools of cytoplasmic and nuclear substrates might gradually convey distinct functions depending on the duration of MAPK activities. Similarly, new factors such as scaffolds might modify the interaction and kinase properties of MPK3/6, allowing the phosphorylation of new proteins or new sites. If MAPK activations last for several hours or are repeated at rapid intervals in the same cell populations, it is also likely that the sites with the most affinity for the kinases would reach saturation, and that new sites with less affinity would be targeted, possibly giving rise to new functions. The proof of this concept was recently adduced by a study in mammalian cells, which demonstrated, using nuclear magnetic resonance spectroscopy, that the MAPK ERK2 phosphorylates in vitro eight sites on the transactivation domain of its serum response factor partner Elk-1, with markedly different rates, unveiling the existence of fast (phosphorylated within minutes) and slow (phosphorylated after several hours) phosphoacceptors. What is more, the authors showed in vivo that whereas fast phosphorylations enhance the Elk-1 transcriptional activity, slow phosphorylations have the opposite effect, suggesting that the sustained activation of MAPK can allow the emergence of new protein functions—in this precise case, a self-limiting function [119]. Interestingly, in *Arabidopsis*, the Et-related EIN3 transcription factor harbors two S/TP sites which are responsible for two contrasting phenotypes: in protoplast assays, the Thr174 is required for the stability of the protein, while the Thr592 promotes its degradation [120]. Although the temporal dynamic of EIN3 phosphorylation by MAPK was not directly addressed, the evidence that only the phosphorylation by MPK3/6 of Thr174 could be detected in vitro or in protoplasts [120], and that Et signaling can be associated with sustained activations of MPK3/6 [50], is compatible with EIN3 functions being modulated by MPK3/6 phosphorylation on two different sites with two different kinetics. Further experiments are needed to confirm this hypothesis, and notably to rule out the possibility that Thr592 is phosphorylated by other kinases than MPK3/6. In the same order of ideas, it appears that multiple sites of phosphorylation by MAPKs are often clustered in the substrate sequences, suggesting some additive or possibly synergistic effects between them. For instance, the phosphorylations of ACS6 on Ser480, Ser483 and Ser488 endow the protein with a negative charge, which inhibits its degradation by the proteasome, and this inhibition can be enhanced by adding more negative charge in the areas surrounding the MAPK phosphorylation sites [112]. Investigating the temporal pattern of clustered MAPK phosphorylation sites should thus offer a better understanding of how sustained MPK3/6 activities might control dose-dependent responses. Collectively, the data we discussed above concur to make the temporal MPK3/6 phosphocode relevant with respect to ETI regulation. However, for validation, significant efforts remain to be made, and new dedicated tools or protocols should be developed so as to comprehensively embrace these questions.

Under this scheme, we will briefly present three recent phosphoproteomic analyses whose aims were to identify proteins phosphorylated either by sustained MPK3/6 activities or during ETI responses. In Kadota et al. [90], a comparative analysis of the phosphoproteome bound to the plasma membrane in one-month-old *Arabidopsis* leaves expressing the effector AvrRpt2, for 0 h, 1 h and 3 h, revealed 84 sites (corresponding to 49 different proteins) with increased phosphorylation, and 25 sites (corresponding to 23 different proteins) with decreased phosphorylation in the combined 1 h and 3 h timepoints, as compared to the 0 h condition. Albeit not their main focus, the authors noticed that among the 84 sites with increased phosphorylation, 32 (corresponding to 25 different proteins) were S/TP sites, suggesting that MAPKs could be significant drivers of the changes in the membrane-associated phosphorylation landscape during ETI. In Hoehenwarter et al. [121], a phosphoenrichment procedure performed on the total protein extracts from 12-day-old seedlings expressing NtMEK2^DD^ for 6 h allowed the identification of 249 phosphorylated S/TP sites, corresponding to 141 putative substrates of MPK3/6. At last, in Lassowskat et al. [62], a time-course analysis of the phosphoproteomes from six-week-old leaves expressing PcMKK5^DD^ during 4 h, 5 h, 7 h and 8 h, performed comparatively with plants expressing an inactive form of PcMKK5, was carried out. By combining the four timepoints, 201 different phosphosites were identified as S/TP sites, corresponding to 145 putative substrates of MPK3/6. Remarkably, among these 145 candidates, some are phosphorylated already at 4 h, while others appear only at later timepoints, suggesting a progressive modification of the phosphoproteome driven by MPK3/6 in line with the ideas exposed beforehand. Based on gene ontology classifications, the early substrates were enriched in functions related to cellular trafficking, such as nucleocytoplasmic shuttling and vesicle-mediated transport, while the late substrates were enriched in proteins associated with chemical defense responses such as IGS production or the PEN pathway [62,122]. Interestingly, these analyses not only conform to previous observations linking sustained MPK3/6 activities with vesicle transport and phytoalexin accumulation during ETI, but they also imply that the coordination of these functions during ETI would hinge on the temporally-distinct scope of substrates. Surprisingly, when we compared the three phosphoproteomes, we found just three proteins in common: AT1G28280 (MVQ1) (an already characterized substrate of MPK3/6, whose phosphorylation in a PTI context leads to the degradation of the protein [123]), AT2G43680 (IQD14) (a protein with a calmodulin-binding domain), and AT4G28080 (REC2) (a tetratricopeptide repeat-like protein involved in chloroplast formation). The small number of common proteins might be explained by important differences in the experimental procedures, like different plant ages/tissues, different protocols of protein extraction/enrichment, or different types of conditional treatment (NtMEK2^DD^, PcMKK5^DD^ and AvrRpt2). Regardless, these three common proteins, and especially REC2 because of its role in choloroplast metabolism, are promising candidates for classification as specific MPK3/6 substrates during ETI, and deserve further attention. Moreover, by looking more closely at these three proteins, it appears that two of them, MVQ1 and REC2, have multiple S/TP phosphorylation sites that display distinctive dynamic patterns over time [62], suggesting that these putative substrates could encompass slow and rapid phosphoacceptors. In summary, despite certain limitations, like the possible disappearance of the phosphorylated proteins due to destabilization, or the fact that late phosphorylation events might be indirect effects, comparative and time-course phosphoproteomic analyses are powerful tools to investigate in vivo the temporal MPK3/6 phosphocodes, and offer exciting opportunities to answer questions regarding the ETI-specific substrates of these kinases. 

## 6. Conclusions

The roles of MAPK signaling during PTI have been relatively well characterized. By contrast, information regarding their involvement during ETI is quite sparse. However, a body of recent research has disclosed interesting new data that have significantly modified our view of this topic. For instance, both virulent and guarding mechanisms, through which MAPK signaling can respectively be inhibited by or preserved from effectors, have been recently described, and the further analysis of pathogen effectomes or the exploitation of *mkk1mkk2* suppressor screens should continue to decipher the intricate networks of MAPK targeting and surveillance. 

Similarly, the finding that pathogen effectors can provoke a sustained MPK3/6 activation that is atypical compared to the transient activation observed in PTI raises challenging questions that remain, at least to a certain extent, unanswered. First of all, the signaling pathways regulating the sustained MPK3/6 activities are still poorly understood. To elucidate them, the consistent testing of MPK3/6’s responses to diverse effectors should be performed, and efforts in epistatic studies need to be made. The forthcoming single cell analysis of MAPK activities and MAPK-dependent responses will also be important in order to validate the model according to which sustained MPK3/6 activities are achieved through a feedback regulatory loop involving SAR actors that repeatedly provoke transient activations in different cell populations.

Another stimulating question pertains to the functions of sustained MPK3/6 activities. A common conception, to which we referred to several times in this review, is that ETI responses amplify PTI responses by prolonging them over time. In this regard, sustained MPK3/6 activities would fulfil the same functions as transient activities in a prolonged and cumulative way. Nevertheless, a clear demonstration of such a model has not been provided till now, and it is very likely that, as compared to transient activities, durable MPK3/6 activities should lead not only to cumulative outcomes, but also to qualitatively different responses. Future challenges involve the better understanding of how sustained MPK3/6 activities reinforce responses mediated by transient activities, and also how they might promote the emergence of new, ETI-specific and more robust functions.

In line with the previous consideration, the question of ETI-specific MPK3/6 substrates faces difficulties in terms of the possibility of discriminating between substrates of transient and sustained MPK3/6 activities. If a few substrates of MPK3/6 might be involved in ETI responses, it is not clear for the moment whether their roles can be ascribed to a specific phosphorylation status or not. In this sense, ETI context provides a conceptual framework to further assess the temporal phosophocode of MPK3/6, and address the fundamental problems related to the scope of kinase substrates according to the kinetics of kinase’s activities. In addition to this concern, a recent report revealed that the transcription factor WRKY33 can be phosphorylated by both MPK3/6 and the Calcium-Dependent Protein Kinases (CDPK) 5 and 6 on different sites, with different effects on the transactivation activity and the DNA-binding ability of the protein [124]. This finding indicates that, in actual fact, the functions of ETI-specific MAPK substrates could be modulated not only by the temporal pattern of phosphorylation, but also by the actions of other kinases. In this domain, the further elucidation of the MAPK/CDPK phosphocodes should also extend our understanding of plant immune responses.

## Supplementary Materials

Supplementary Materials can be found at https://www.mdpi.com/1422-0067/21/21/7954/s1. Table S1: GO analysis of genes commonly regulated by Pma-AvrRpt2 and NtMEK2^DD^, or MKK4^DD^, or K3CA.

## Abbreviations

CNL	Coiled-Coil NLR
Et	Ethylene
ETI	Effector-Triggered Immunity
HR	Hypersensitive Response
IGS	Indole Glucosinolate
MAPK	Mitogen-Activated Protein Kinases
NLR	Nucleotide-Binding Leucine-Rich Repeat Receptors
PAMP	Pathogen-Associated Molecular Patterns
PRR	Pattern Recognition Receptor
PSII	PhotoSystem II
PTI	PAMP-Triggered Immunity
R	Resistance
RLCK	Receptor-Like Cytoplasmic Kinase
ROS	Reactive Oxygen Species
SA	Salicylic Acid
SAR	Systemic Acquired Resistance
SIPK	SA-induced Protein Kinase, homologue of MPK6
SUMM	Suppressor of *mkk1mkk2*
TMV	Tobacco Mosaic Virus
TNL	Toll Interleukin-1 Receptor NLR
WIPK	Wounding-Induced Protein Kinase, homologue of MPK3
WT	Wild Type
